# The combination of intraoperative CT navigation and C-arm fluoroscopy for INFIX and percutaneous TITS screw placement in the treatment of pelvic ring injury: technical note

**DOI:** 10.1186/s13018-022-02920-0

**Published:** 2022-01-15

**Authors:** Akihiko Hiyama, Taku Ukai, Satoshi Nomura, Masahiko Watanabe

**Affiliations:** Department of Orthopaedic Surgery, Surgical Science, Tokai University School of Medicine, 143 Shimokasuya, Isehara, Kanagawa 259-1193 Japan

**Keywords:** Internal pelvic fixator, Transiliac–transsacral screw, Pelvic ring fracture, Intraoperative CT navigation, C-arm fluoroscopy, Pelvic ring injury

## Abstract

**Background:**

The subcutaneous screw rod system, commonly known as the internal pelvic fixator (INFIX), is useful in managing unstable pelvic ring fractures. Conventional INFIX and transiliac–transsacral (TITS) screw techniques are performed using C-arm fluoroscopy. There have been problems with medical exposure and screw insertion accuracy with these techniques. This work describes new INFIX and TITS techniques using intraoperative computed tomography (CT) navigation and C-arm fluoroscopy for pelvic ring fracture.

**Methods:**

A typical case is presented in this study. An 86-year-old woman suffered from an unstable pelvic ring fracture due to a fall from a height. INFIX and TITS screw fixation with intraoperative CT navigation were selected to optimize surgical invasiveness and proper implant placement.

**Results:**

The patient was placed in a supine position on a Jackson table. An intraoperative CT navigation was imaged, and screws were inserted under the navigation. Postoperative X-rays and CT confirmed that the screw was inserted correctly. This technique was less invasive to the patient and had little radiation exposure to the surgeon. Rehabilitation of walking practice was started early after the surgery, and she was able to walk with the assistance of a walker by the time of transfer.

**Conclusions:**

The technique employed in our case study has the cumulative advantages of safety, accuracy, and reduced radiation exposure, together with the inherent advantages of functional outcomes of previously reported INFIX and TITS screw techniques. Further experience with this approach will refine this technique to overcome its limitations and facilitate its wider use.

**Supplementary Information:**

The online version contains supplementary material available at 10.1186/s13018-022-02920-0.

## Background

Pelvic ring fractures are commonly caused by high-energy trauma due to a car accident or a fall from a height. Fracture patterns determine conservative and surgical treatment [1]. Severe unstable pelvic ring fractures, such as type C in the AO Foundation and Orthopedic Trauma Association (AO/OTA) classification, are usually treated surgically. The OTA and AO Foundation have adopted the imaging-based Tile classification system for pelvic instability grading [2, 3].

To our knowledge, there are three types of internal fixation methods to be considered in the stabilization of pelvic ring fractures: anterior plate placement using an ilioinguinal approach; iliosacral (IS) screw fixation; and posterior lumbopelvic fixation using pedicle screw systems. In recent years, advances in spinal implants have allowed a minimally invasive spinal treatment (MIST) procedure for posterior pelvic ring fractures to be developed [4–6]. A recent systematic review concluded that posterior pelvic internal fixation might yield better clinical results than nonoperative treatment and stabilization of the anterior pelvis [7]. However, surgical exposure to posterior fixation is associated with a high wound complication rate. In particular, severe wound complications such as skin necrosis may occur when patients are treated with transcatheter arterial embolization [8, 9].

Based on this background, the subcutaneous pedicle screw–bar fixation (INFIX) procedure, an anterior procedure using a spinal instrument, has been reported [10, 11]. INFIX is a minimally invasive osteosynthesis technique with potential benefits, such as fewer soft tissue infections, better pain control, better patient mobilization, and faster rehabilitation. This procedure could be of potential use in complex fracture patterns of the anterior pelvic ring and reduce morbidity due to its decreased intraoperative time and blood loss compared with conventional techniques [12].

Unfortunately, intraoperative C-arm fluoroscopy is repeatedly taken during the procedure for positioning the screw and the level of radiation exposure to both the patient and surgical staff is extensive. In addition, the obtained images are blurred and cannot clearly show the anatomical structure around the sacroiliac joint. This is especially the case of anatomical variation where the rate of screw malpositioning is increased, adding considerably to the stress of the surgeon and the surgical team.

In recent years, there have been some reports of spinal surgery using surgical support devices, such as intraoperative computed tomography (CT) navigation and robots, to solve the problem of C-arm fluoroscopy [13–16]. Similarly, intraoperative CT navigation systems have been introduced for the screw fixation of pelvic ring injuries [17, 18]. The use of image-guided instrumentation allows a high degree of surgical precision together with a significant reduction in ionizing radiation production within the operating room. In this technical note, we describe a novel technique for treating pelvic ring fractures using the intraoperative CT navigation system with an INFIX technique and transiliac–transsacral (TITS) screw fixation to reduce intraoperative C-arm fluoroscopy.

## Methods

Under the intraoperative CT navigation system, we will explain the techniques for the pelvic ring fractures using the INFIX technique and TITS screw fixation.

### INFIX approach using intraoperative CT navigation

Stabilization of the anterior pelvic ring was performed using intraoperative CT navigation in a manner based on the INFIX technique described by Vaidya et al. [19].

**Fig. 1:**
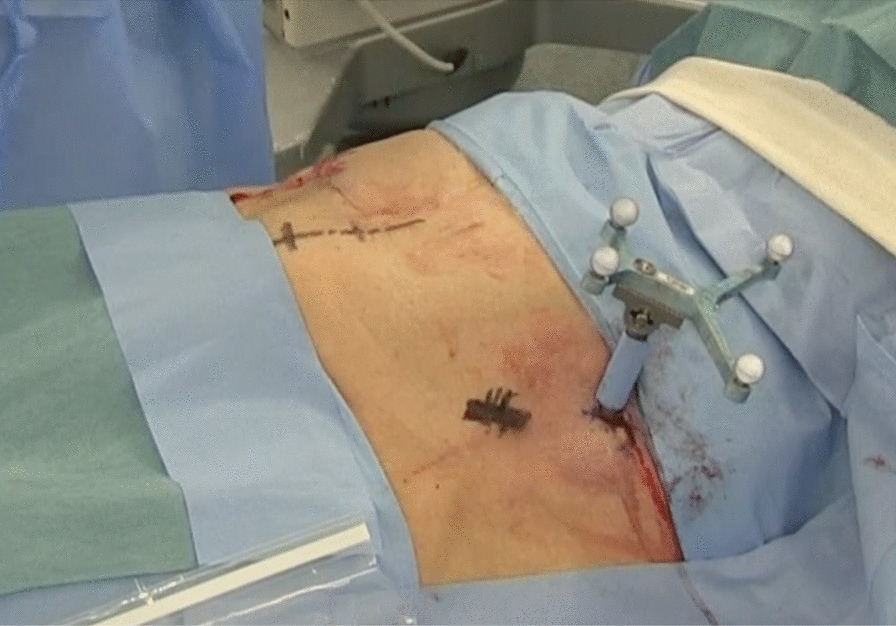
Intraoperative images of the percutaneously placed reference pin and attached navigation frame in the iliac crest

The patient was operated on under general anesthesia based on the anesthetists’ decision. She was placed in a supine position on the operating carbon table (OSI Modular Table System; Jackson table; Mizuho, Union City, CA, USA) for the CT scan by O-arm (O-arm2® imaging system, Medtronic plc, Dublin, Ireland). Standard spine instruments and navigated spinal instruments were used for the surgery. After proper painting and draping, the navigation reference frame was fixed to the iliac crest (Fig. 1). Then, the O-arm was positioned, and the 3D-reconstructed images were obtained and transmitted to the StealthStation surgical navigation system (S7; Medtronic Sofamor Danek, Minneapolis, MN, USA). The relative spatial position of the patient's tracker and pelvis should not change during surgery.

Navigation was confirmed, and skin markings were made to identify the anterior superior iliac spine and pubic symphysis. After every navigated spinal instrument was verified, the best entry point on the anterior inferior iliac spine (AIIS) was marked by the navigated pinpoint probe.

Through a 2–3 cm vertical incision centered on the AIIS, the entry point on the AIIS was approached by careful dissection through the interval between the sartorius and the tensor fascia lata, taking utmost care to protect the lateral femoral cutaneous nerve.

**Fig. 2:**
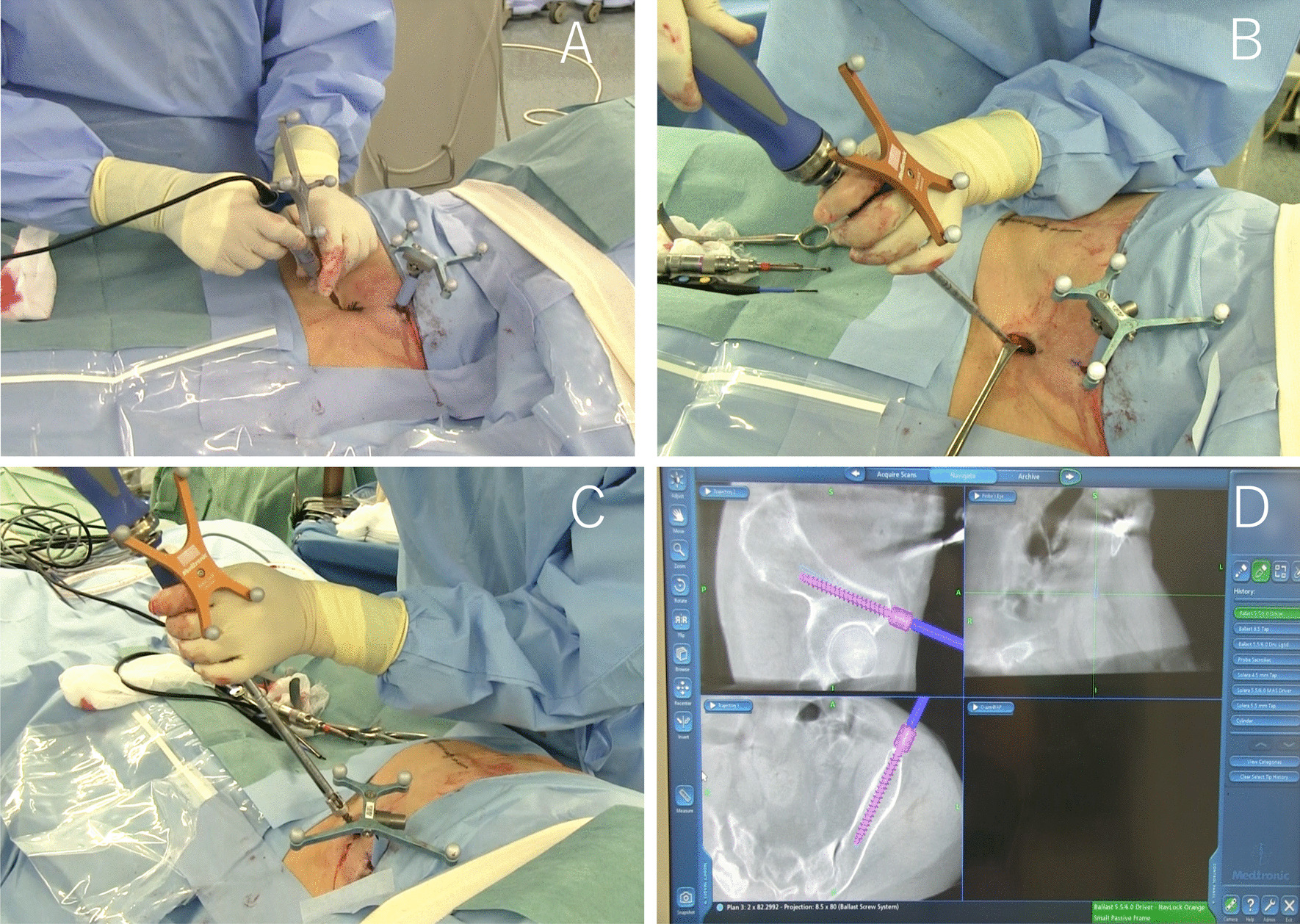
INFIX technique using intraoperative CT navigation. Navigated high-speed burr Stealth–Midas System™ (**a**). Navigated pedicle tap (**b**). Navigated CD Horizon® Ballast™ screw (**c**). StealthStation computer screen projection of a Ballast™ screw being inserted into the iliac crest with a navigated driver (**d**)

The navigated high-speed burr Stealth–Midas System™ (Medtronic Sofamor Danek), pedicle probe and tap were used to make the screw hole, and then a CD Horizon® Ballast™ screw (Medtronic Sofamor Danek) was inserted (Fig. 2).

### Screw implant selection

In the present study we used the Ballast™ screw, which is a polyaxial head screw. The ring notch at the base of the Ballast™ screw head provides a swing angle of up to 40°, making it easy to connect the rods. While looking at the virtual line on the navigation monitor, the Ballast™ screw was advanced from the AIIS. Even though it decreases the overall strength, it provides greater adjustments for the placement of the rod to decrease abdominal impingement.

Navigation was also used to size the screw according to the patient’s physique, but most surgeons use multiaxial pedicle screws with a diameter of 8.5 mm and a length of 80 mm, with at least about 60 mm of the screw being intraosseous and about 20 mm outside the bone. We ensured that the pedicle screw head remained above the deep fascia to avoid compression on the femoral nerve and inguinal ligament.

**Fig. 3:**
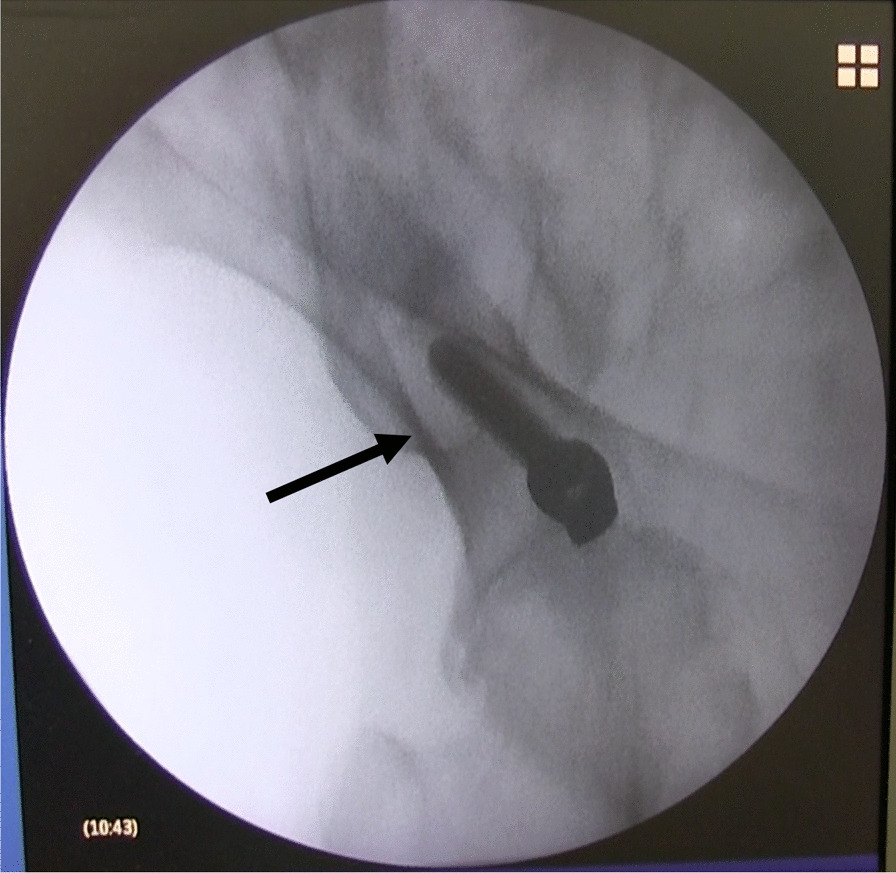
Fluoroscopic image. Obturator oblique view showing the position of Ballast™ screw (arrow)

To confirm the screw placement, the C-arm fluoroscopy was adjusted to show the iliac view, and the awl was advanced directed towards the ischial spine (Fig. 3).

### Rod selection and formation

**Fig. 4:**
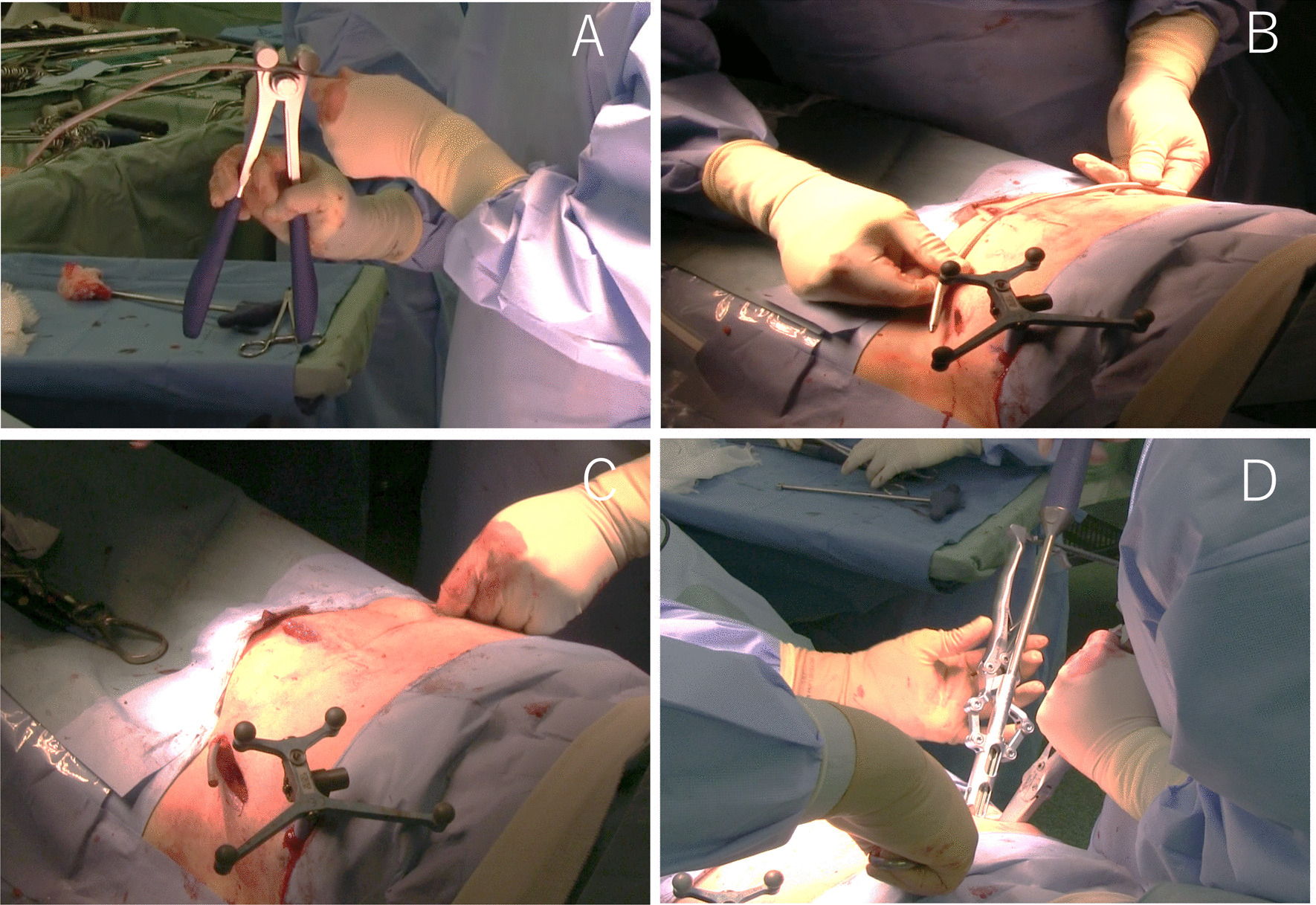
INFIX technique. Overcontoured rod (**a**). Rod formation (**b**). Rod being tunneled subcutaneously (**c**). Tightening the Ballast™ screw (**d**)

The required rod length was measured and the rods were bent in semblance with the contour of the anterior pelvis. A 5.5-mm titanium rod was tunneled subcutaneously, connected to Ballast™ screw heads, and locked at one end (Fig. 4). The rod was inserted from one side and guided gently in the subcutaneous plane towards the other side. Once the rod was near the contralateral screw, it was guided onto the opposite pedicle screw using rod-holding forceps. The blocker was locked onto the Ballast™ screw head on one side once the rod was in position.

Reduction of the anterior pelvic ring injury was achieved by compression or distraction over rods as appropriate prior to locking the remaining screw head. The reduction was also done by distraction/lateral compression of the pelvic ring/traction and internal rotation.

The quality of reduction and implant position was confirmed on C-arm fluoroscopy anteroposterior, inlet, and outlet views. The final tightening was done after confirming a satisfactory reduction. Finally, the position of the rod was checked by inserting two fingers between the rod and bone. At least 1 cm of the rod was kept proud on each side to help in removal.

We present a video about the INFIX technique with intraoperative CT navigation (Additional file 1: Video 1).

### Placement of IS or TITS screws

The fixation of IS screws including the TITS screw placement under C-arm fluoroscopy is a technically demanding procedure, which includes multiple fluoroscopic confirmations due to complex posterior pelvic structures and a high degree of upper sacral variability.

**Fig. 5:**
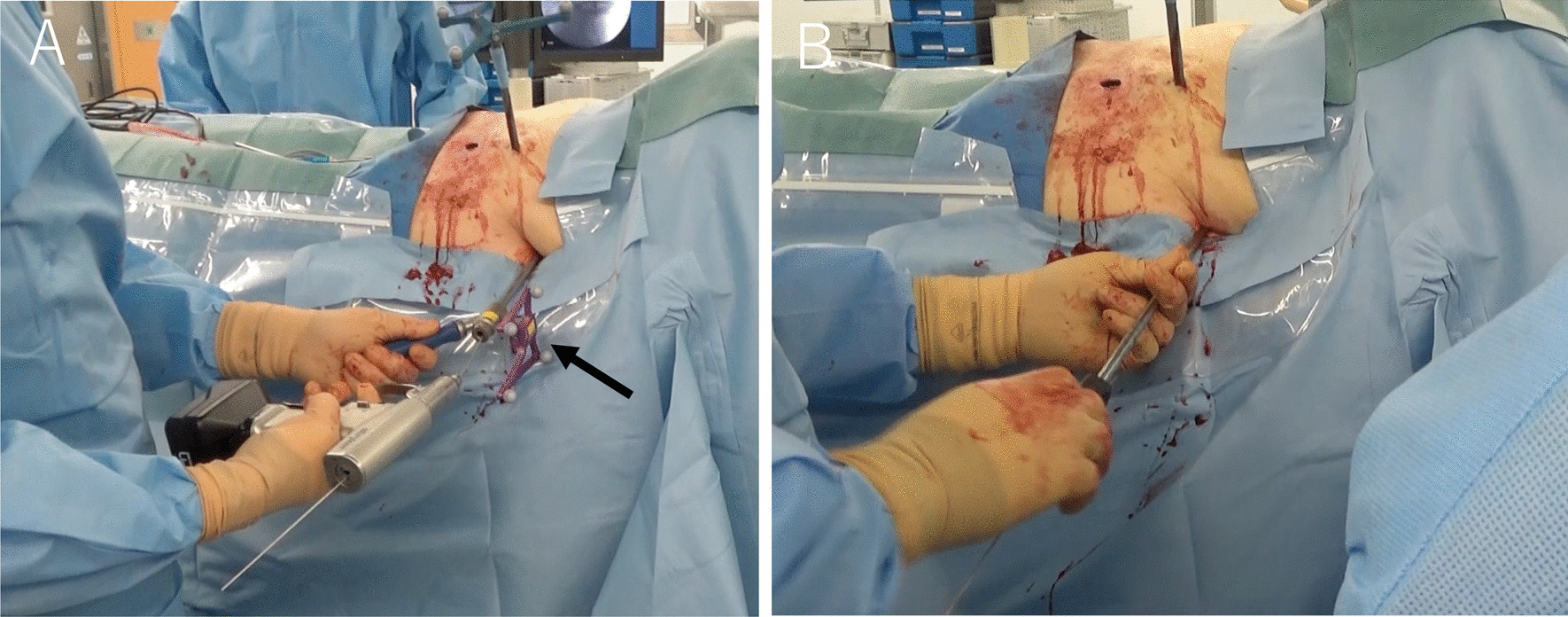
Transiliac–transsacral (TITS) screw navigation for pelvic ring fracture. The guide wire was inserted into the starting point on the fracture side using intraoperative CT navigation and Universal Cannulated Screw Set (UCSS, Medtronic) (**a**). The arrow indicates Universal Cannulated Screw Set. Under fluoroscopy and navigation, we confirmed that the guide wire was inserted into the iliac sacrum. We inserted the Transiliac–transsacral (TITS) over the guidewire (**b**).

**Fig. 6:**
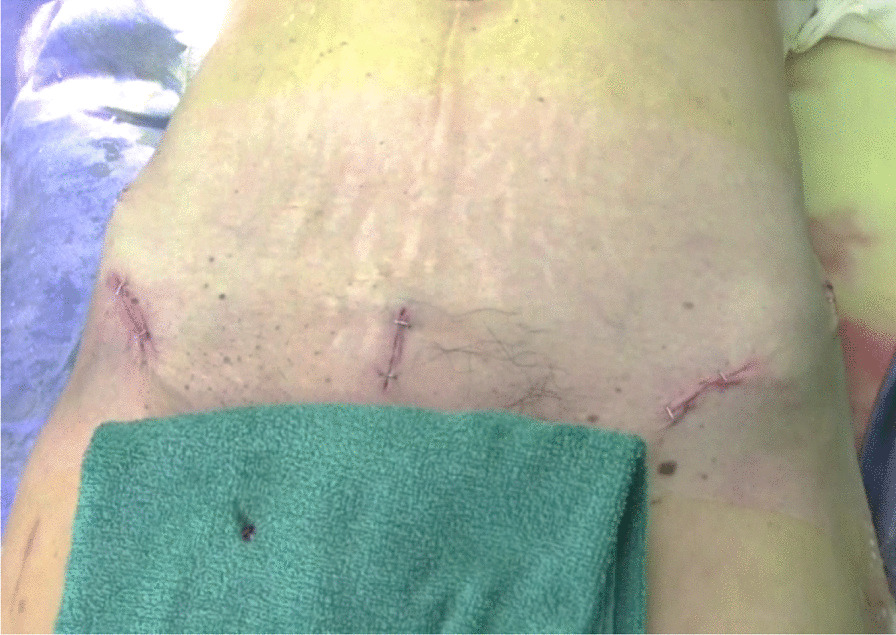
Intraoperative image of the INFIX incision

In our procedure, percutaneous screw fixation using 6.5 mm diameter titanium cannulated cancellous screw (CCS) (Meira, Nagoya, Japan) was performed with intraoperative CT navigation together with C-arm fluoroscopy depending on the morphology and displacement of the sacrum (Fig. 5). CCS fixation is usually applied to the ipsilateral side of the sacrum. Our approach was with the navigated Universal Cannulated Screw Set (UCSS, Medtronic) using the hollow to insert the guide wire. The navigated cannulated pedicle awl was moved until the direction of the cannulation was completely consistent with the planned screw position. The position of the virtual cannulated line on the coronal, sagittal, and cross section of the target segment was observed on the screen. The direction is prepared by placing the navigated cannulated pedicle awl over the guide wire and twisting it into the iliac and sacrum. After that, we used a cannulated drill tap to make a screw hole. The guide wire was held in position when removing the precision tap. CCSs were placed and tightened sequentially through the inserted guide wire, and finally X-rays were obtained. In cases of bilateral or transverse sacral fractures, fixation is performed bilaterally using a TITS screw method. Many external fixators were removed preoperatively. However, the reduction position is examined and it is decided whether to remove it during the operation. The skin incision by this procedure is minimal (Fig. 6). We present a video about the IS screws techniques including the TITS screw placement (Additional file 2: Video 2).

Although the patients' walking start time varies, we allowed our patients to ride in a wheelchair and walk according to pain from the day after the operation during the early postoperative period.

## Results

### Patient history

**Fig. 7:**
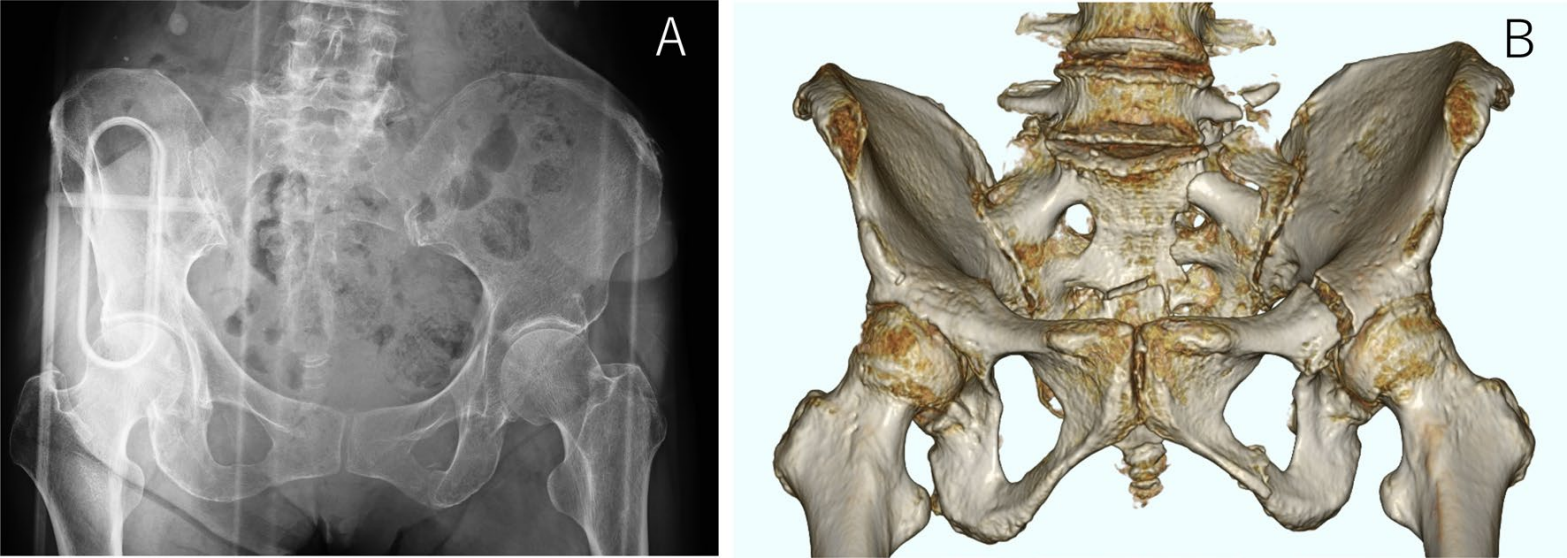
Preoperative X-rays and computed tomography (CT). Pelvic anteroposterior (AP) view (**a**). Preoperative three-dimensional CT reconstruction of AP view (**b**)

An 86-year-old woman with dementia was hospitalized following a fall from a height. Her diagnosis was a pelvic ring fracture (C1–3 type) with a sacral fracture and a bilateral upper and lower fracture of the pubis. Her preoperative X-ray and three-dimensional (3D) CT are shown in Fig. 7.

The day after the injury, INFIX and TITS fixation using intraoperative CT navigation was performed for the pelvic ring fracture. The operation time was 161 min and the estimated blood loss was 137 mL. In terms of radiation exposure, the patient organ doses were 0.140 mGy in the neck, 0.287 mGy in the chest, and 2.675 mGy in the abdomen measured using a dosimeter. In addition, a chest dosimeter inside the surgeon's protector recorded 0.018 mGy.

**Fig. 8:**
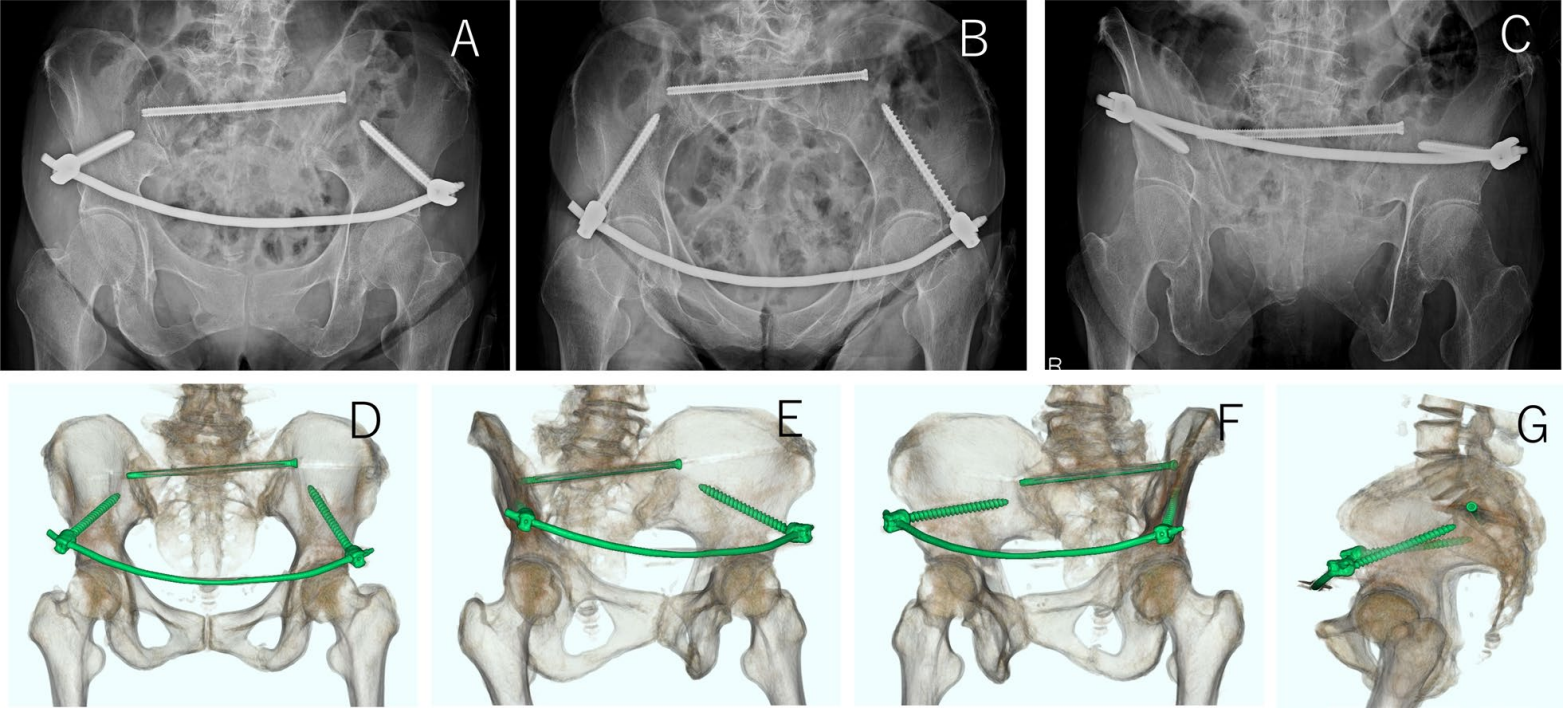
Postoperative X-rays and CT. X-rays; Pelvic AP view (**a**), Pelvic inlet view (**b**), and Pelvic outlet view (**c**). Postoperative three-dimensional CT reconstruction; AP view (**d**), Obturator oblique view (**e–f**), and lateral view (**g**)

A postoperative X-ray and 3D CT scan showed that the screws were in the correct position after the operation (Fig. 8). The patient was transferred to rehabilitation 37 days after surgery. Her walking level at the time of transfer to another hospital was assisted walking using a walker.

## Discussion

Unstable pelvic fractures often require concurrent stabilization of the anterior and posterior pelvic rings. In addition, symphyseal plating leads to more blood loss when compared with INFIX [20]. This is because plating requires exposing the fracture site and the whole process of peeling of soft tissues and dissection around the area, which is rich in blood vessels and leads to greater intraoperative bleeding. In addition, open reduction and internal fixation (ORIF) of comminuted anterior pelvic ring fractures is associated with extensive soft tissue damage and increased incidence of surgical site infection and wound complications, risk of neurovascular and visceral injury, surgical site hernia, and implant failure. However, the stability of the posterior pelvic ring can be fully restored by MIST, such as a lumbopelvic fixation compared to ORIF in the anterior direction [21]. Even so, there is concern about the possibility of low back pain after surgery because the operation remains invasive to the posterior element.

INFIX is one method that uses the already established principle of external fixation and is useful for pelvic ring fractures. Biomechanical studies have shown that INFIX has superior stability with respect to axial stiffness and stiffness at the pubic symphysis in the management of vertically and rotationally unstable pelvic ring injuries [12]. According to a systematic review of conventional INFIX, most surgeons place screws using a mini-open or percutaneous method under strict fluoroscopic guidance [12, 22]. The implants used were pedicle screws and rods of various spinal systems. With conventional INFIX, the rod is contoured and placed subcutaneously. Then, after reducing fracture by different indirect mechanisms and checking reduction under fluoroscopic guidance, the rod is fixed with screws. Screw lengths and thicknesses vary from study to study and from patient to patient and usually require longer screws. Bone quality is also considered, but the most common screws are 6.5–7 mm diameter and 55–120 mm in length. The Ballast™ screw we used has a diameter of 8.5 mm, and we have selected screws with a length of 80 mm or more. The rods used are reported to be 5 to 6.5 mm in diameter, but we used 5.5 mm titanium rods. If necessary, posterior fixation is usually attempted. We first performed the anterior fixation with INFIX in our patient and then performed posterior fixation with IS or TITS screws. The reason for this is that we believe that it has benefits for pelvic reduction.

The technique of conventional INFIX and TITS screws using C-arm fluoroscopy entails radiation exposure. In addition, multiplanar repeated images are necessary for their accurate insertion, which increases radiation and operating time. On the other hand, in previous studies, intraoperative CT showed the highest screw accuracy at the screw position, but intraoperative CT navigation led to significantly longer total procedure and fluoroscopic times. Moreover, the patient’s radiation dose was significantly higher compared to that under C-arm fluoroscopy. It has also been reported that the irradiation dose is significantly higher than that of C-arm fluoroscopy [23]. Our study shows that the combination of intraoperative CT navigation and C-arm fluoroscopy eliminates the need for cumbersome lead gears and reduces surgeon’s radiation exposure without compromising accuracy compared to surgical operations with C-arm fluoroscopy alone. Thus, our technique employed for pelvic ring fractures using intraoperative CT navigation has the cumulative advantages of safety, accuracy, shorter operating time, and reduced radiation exposure, together with inherent advantages of better fusion rates and functional outcomes of INFIX and IS or TITS screw techniques.

There are still some limitations of this study. First, the surgeon's learning curve can affect the techniques. In addition, we did not compare the radiation exposure or accuracy of screw insertion with INFIX and TITS techniques to traditional fluoroscopy-only procedures in this study. Finally, this paper is a technical note, not an original article, and it may be necessary to evaluate the usefulness of this procedure in the future. All these limitations should be addressed in the future. To confirm the advantages of this method, a prospective multicenter randomized controlled trial compared with the current standard of care will be necessary.

## Conclusion

The INFIX and TITS screw technique can be considered a viable alternative to ORIF for unstable pelvic ring injuries as discussed in previous systematic reviews. In addition, we showed that our reported technique might be able to improve screw insertion accuracy and reduce medical radiation exposure.

## Supplementary Information

**Additional file 1:** INFIX technique with intraoperative CT navigation.

**Additional file 2:** TITS technique with intraoperative CT navigation and fluoroscopy.

## Data Availability

Data are available upon request from the corresponding author.

## Abbreviations

INFIX: Internal pelvic fixator
TITS: Transiliac–transsacral
IS: Iliosacral
MIST: Minimally invasive spinal treatment
AIIS: Anterior inferior iliac spine
CCS: Cannulated cancellous screw
ORIF: Open reduction and internal fixation
